# Supplementary material for: Structural and Energetic Affinity of Annocatacin B with ND1 Subunit of the Human Mitochondrial Respiratory Complex I as a Potential Inhibitor: An In Silico Comparison Study with the Known Inhibitor Rotenone
Source: Polymers (Basel). 2021 Jun 2;13(11):1840. doi: 10.3390/polym13111840 (PMC8199665; doi:10.3390/polym13111840)
Supplement: Supplementary file 1 [file polymers-13-01840-s001.zip › polymers-1223028-SI.pdf]

Structural and Energetic Affinity of Annocatacin B with ND1 Subunit of the Human Mitochondrial Respiratory Complex I as a Potential Inhibitor: An in silico Comparison Study with the Known Inhibitor Rotenone

May 27, 2021

Camilo Febres-Molina[1]; Jorge Alberto Aguilar-Pineda[2]; Pamela Gamero-Begazo[1]; Haruna L. Barazorda-Ccahuana[1]; Diego E. Valencia[1]; Karin J. Vera-López[2]; Gonzalo Davila-Del Carpio[3], Gómez Valdez, Badhin[1,4*]

[1]Centro de Investigación en Ingeniería Molecular, CIIM, Universidad Católica de Santa María, Urb. San José s/n - Umacollo, Arequipa Perú.
[2]Laboratory of Genomics and Neurovascular Diseases, Vicerrectorado de Investigación, Universidad Católica de Santa María, Arequipa, Peru.
[3]Vicerrectorado de Investigación, Universidad Católica de Santa María, Arequipa Perú.
[4]Facultad de Ciencias Farmacéuticas, Bioquímicas y Biotecnológicas, Universidad Católica de Santa María, Urb. San José s/n - Umacollo, Arequipa, Perú

Keywords: Annocatacin B, ND1 subunit, mitochondrial respiratory complex I, MRC-I, molecular dynamics simulations, MD, Hirshfeld charges, MM/PBSA

[*]Author to whom correspondence should be addressed.
Electronic mail: bgomez@ucsm.edu.pe; Tel.: +51-982895067

Supplementary Figures

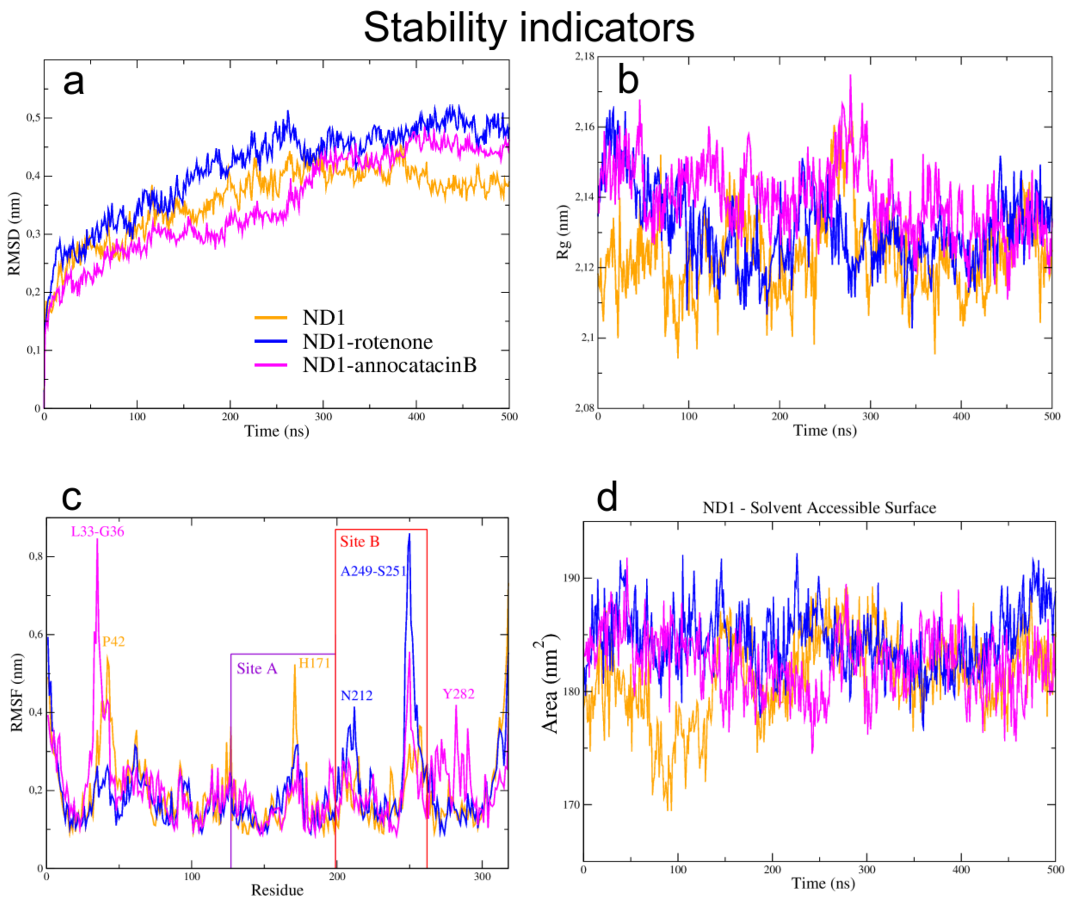

Figure S1. Stability indicators used in this work. Plots were obtained in full MD simulations. (a) Root mean square deviation (RMSD). (b) The total radius of gyration (RG). (c) Root mean square fluctuation of Cα atoms. The A and B sites comprise the active site of the ND1 subunit. (d) Solvent accessible area (SAS) of the ND1 protein in the molecular complexes.

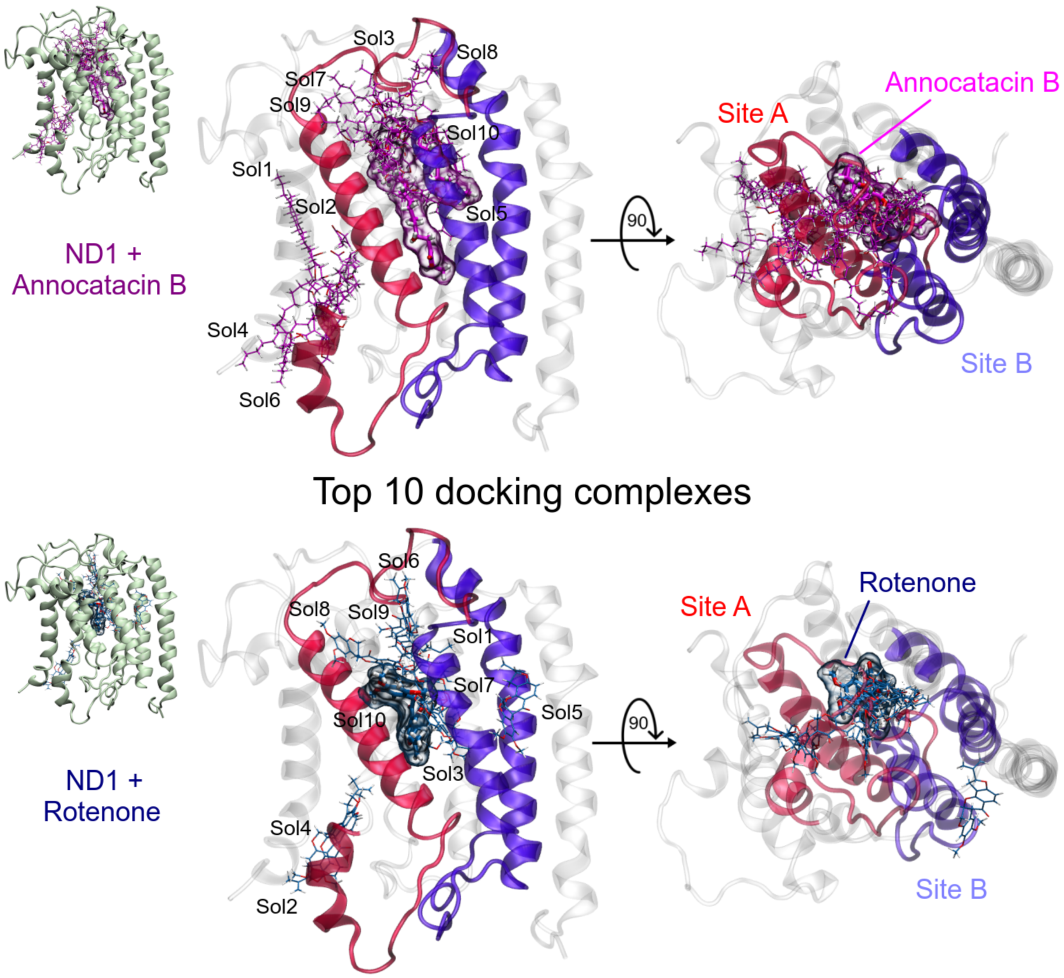

Figure S2. Top 10 structure complexes of the PatchDock docking results. Above figures correspond to the ND1-Annocatacin B complexes. Below figures correspond to the ND1-Rotenone complexes.

Table S1. System details in the MD simulations.

System details

Protein	Aminoacids	DPPC molecules	Ligand	Solvent molecules	Na ion	Total atoms	box size [nm]	Run time [h] (Approx.)
ND1	318 (5127)	507 (25350)	-	37861 (113583)	1	144061	11.70 x 11.74 x 12.98	2496
ND1 + Rotenone	318 (5127)	507 (25350)	1 (51)	43345 (130035)	1	160563	11.64 x 11.68 x 14.34	2976
ND1 + Annocatacin B	318 (5127)	507 (25350)	1 (103)	37817 (113451)	1	143982	11.62 x 11.66 x 13.13	2351
